# Electron beam lithography on nonplanar and irregular surfaces

**DOI:** 10.1038/s41378-024-00682-9

**Published:** 2024-04-19

**Authors:** Chenxu Zhu, Huseyin Ekinci, Aixi Pan, Bo Cui, Xiaoli Zhu

**Affiliations:** Department of Electrical and Computer Engineering and Waterloo Institute for Nanotechnology (WIN), University of Waterloo, Waterloo, ON Canada

**Keywords:** nanofabrication, electron beam lithography, plasma etching, nonplanar and irregular surfaces, Electrical and electronic engineering, Electronic properties and materials

## Abstract

E-beam lithography is a powerful tool for generating nanostructures and fabricating nanodevices with fine features approaching a few nanometers in size. However, alternative approaches to conventional spin coating and development processes are required to optimize the lithography procedure on irregular surfaces. In this review, we summarize the state of the art in nanofabrication on irregular substrates using e-beam lithography. To overcome these challenges, unconventional methods have been developed. For instance, polymeric and nonpolymeric materials can be sprayed or evaporated to form uniform layers of electron-sensitive materials on irregular substrates. Moreover, chemical bonds can be applied to help form polymer brushes or self-assembled monolayers on these surfaces. In addition, thermal oxides can serve as resists, as the etching rate in solution changes after e-beam exposure. Furthermore, e-beam lithography tools can be combined with cryostages, evaporation systems, and metal deposition chambers for sample development and lift-off while maintaining low temperatures. Metallic nanopyramids can be fabricated on an AFM tip by utilizing ice as a positive resistor. Additionally, Ti/Au caps can be patterned around a carbon nanotube. Moreover, 3D nanostructures can be formed on irregular surfaces by exposing layers of anisole on organic ice surfaces with a focused e-beam. These advances in e-beam lithography on irregular substrates, including uniform film coating, instrumentation improvement, and new pattern transferring method development, substantially extend its capabilities in the fabrication and application of nanoscale structures.

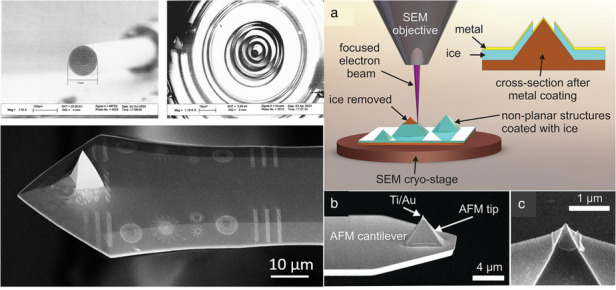

## Introduction

Nanofabrication on nonplanar and irregular surfaces has become an increasingly popular manufacturing technique among researchers due to its wide range of applications in various fields. One of the primary uses of this technique is the fabrication of atomic force microscopy (AFM) tips. These modified tips are utilized in tip-enhanced Raman spectroscopy (TERS) for chemical analysis^1^, magnetic force microscopy, and scanning single electron transistor (SET) microscopy for the study of mesoscopic systems^2,3^. Additionally, AFM cantilevers with metal wires can be used to study fundamental quantum mechanical systems, such as Bose−Einstein condensates^4^ and mesoscopic persistent currents^5^. Another application of this method is the fabrication of optical fibers. An optical fiber with dielectric and metallic nanostructures on its tip can be designed for chemical and biological sensing^6–9^. Moreover, the fabrication of porous and fragile Si_3_N_4_ membranes is promising because these membranes are essential platforms for plasmonic nanostructures^10,11^ and nanopores^12^.

One straightforward method used to pattern irregular surfaces is the focused ion beam (FIB) technique^13,14^, which involves the deposition of a thin film onto the surface of a sample and the direct machining of the film with a high-energy beam. However, FIB is very time-consuming and costly. Nanoimprint lithography (NIL) is another patterning method^15–17^ in which patterns are created by the mechanical deformation of an imprint resist using a template. NIL has a very high throughput, but producing a template for every design is inconvenient. Unlike FIB and NIL, electron beam (e-beam) lithography (EBL)^18^ does not require a template, can easily write quickly, and can expose a thick resist without ion contamination. Thus, EBL is widely used for nanofabrication.

Typically, the EBL process involves resist coating, e-beam exposure, development, and pattern transfer (via direct etching or lift-off). However, the use of resist coatings on irregular substrates or samples is problematic since spin coating only works well on conventional surfaces. Several methods for coating irregular surfaces have been reported, such as resist evaporation^19,20^, spray coating^21^, ice lithography^22,23^, frozen carbon dioxide resist utilization^24^, polymer brush grafting^25,26^, self-assembled monolayer formation^27^ and silicon dioxide resist utilization^28^. In this review, five main methods for achieving EBL on irregular surfaces are explored.

### E-beam lithography using an evaporated resist

In lithography, spin coating of resists works well on flat surfaces. However, for irregular surfaces, alternative resist coating techniques, such as evaporation, are necessary.

During evaporation, the material to be processed is loaded and heated in a high vacuum environment with a pressure of less than 10^−5^ Torr. There are two types of evaporation methods—thermal and e-beam evaporation methods^29,30^—which depend on the method used to heat the source material. Both methods involve line-of-sight deposition, suggesting that any surface, even nonplanar surfaces, can be coated. The source materials used for evaporation must be solid, and researchers have chosen three types of “dry” resists for this process: nonpolymeric sterol (e.g., QSR-5), metal halide (e.g., AlF_3_ and NaCl), and polystyrene.

#### Evaporated nonpolymeric sterol

**Fig. 1:**
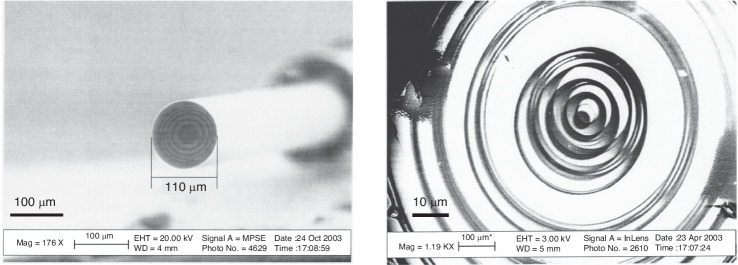
SEM images of the top view of a fabricated zone plate on a fiber tip^33^. Reproduced with permission from AVS (2004)

Currently, PMMA is the preferred resist for e-beam lithography^31,32^. This “wet” resist is applied to the surface of a sample via spin-coating. Conversely, QSR-5 is a “dry” resist composed of nonpolymeric sterol molecules that is applied via evaporation. The deposition method is the only difference between QSR-5 and other resists in the fabrication process. QSR-5, a negative resist that is effective for direct etching and lift-off, was commercialized by Quantiscript Inc. Kelkar et al. successfully fabricated a Fresnel zone plate microlens on the tip of a fiber using an evaporated sterol-based resist^33^. A thin Cr layer was first deposited via evaporation on a fiber tip. Then, the sterol-based resist was evaporated. After e-beam exposure and development, the pattern was transferred to a Cr layer by wet etching. Figure 1 displays the obtained results, with a fiber diameter of 110 µm and a calculated final zone diameter of 99.2 µm.

**Fig. 2:**
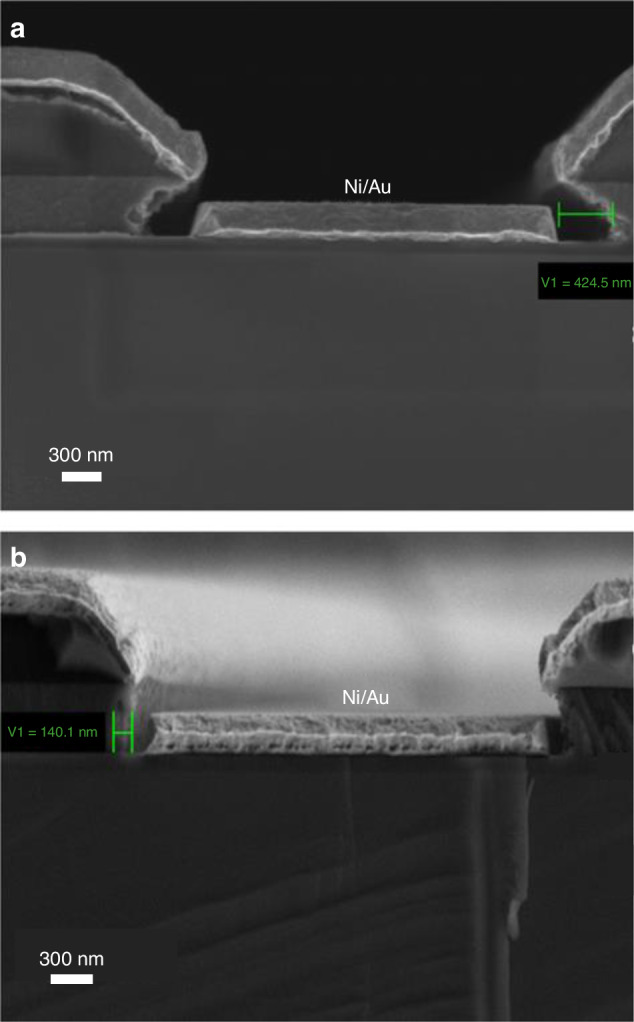
Results of Ni/Au metallization. Cross-sectional SEM images of the samples **a** with and **b** without Ar^34^. Reproduced with permission from Elsevier (2013)

**Fig. 3:**
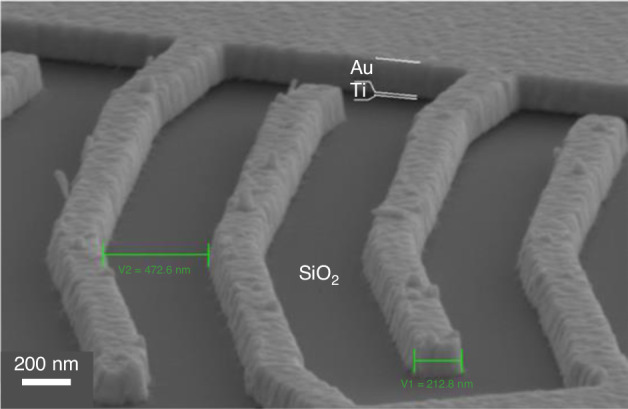
SEM image of the sample after lift-off^34^. Reproduced with permission from Elsevier (2013)

Sterol-based resists are applicable to the lift-off process, as reported by Gerbedoen et al.^34^. To create the desired pattern, these scholars first applied a 100-nm-thick SiO_2_ layer as a stop layer using low-pressure chemical vapor deposition. Next, 450 nm of a-Si was evaporated and QSR-5 was applied for deposition. Lithography was conducted using a scanning electron microscope with a field emission gun at 20 keV and an exposure dose of 5630 µC/cm_2_. After developing with methyl ethyl ketone, two dry etching mixtures listed in Table 1 were used to transfer the pattern to the a-Si layer: one using only SF_6_ and the other using both SF_6_ and Ar. Ni and Au were deposited after dry etching. SEM images of the samples before lift-off are shown in Fig. 2, revealing that the SF_6_-only mixture resulted in a significant undercut, while the mixture with Ar was relatively controllable. The lift-off process was completed with a 20% TMAH solution, producing the desired metal pattern (Fig. 3). In summary, using a sterol-based resist is a novel approach for conducting e-beam lithography on small and irregular surfaces. The process is simple, with the resist coating method being the only notable difference from other common approaches.

**Table 1:** List of two mixtures used to etch a-Si^34^

	Recipe 1	Recipe 2
P_coil_ (W)	600	600
P_platen_ (W)	10	10
Pressure (mTorr)	12	12
Gas (sccm)	SF_6_ (20)	SF_6_/Ar (15/5)
Time (s)	45	45

Reproduced with permission from Elsevier (2013)

#### Evaporated metal halides

**Fig. 4:**
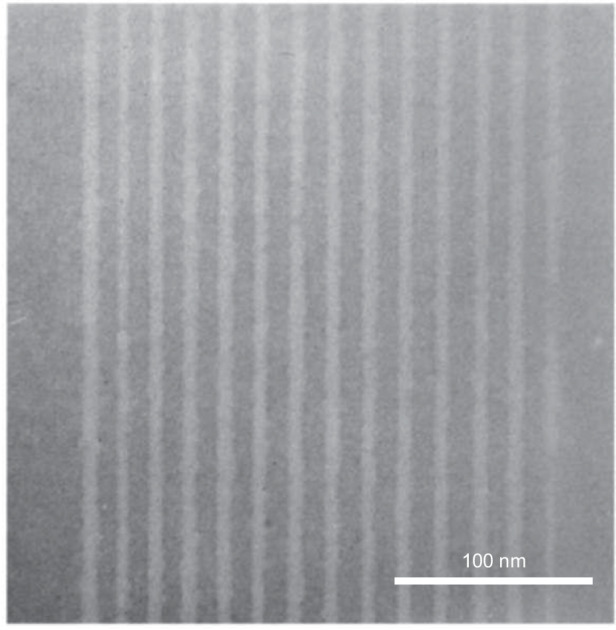
STEM micrograph of a 16-nm-thick aluminum fluoride resists, showing lines with a width of 7 nm and a spacing of 17 nm^37^. Reproduced with permission from Elsevier (1989)

**Fig. 5:**
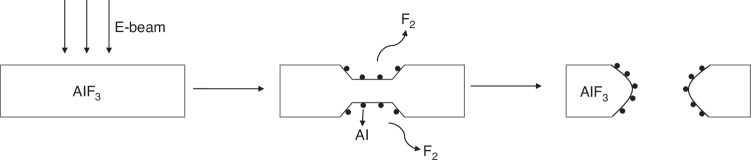
Mechanism of the self-development of metal halides

Metal halides are compounds of metals with halogens^35,36^. During the 1980s, various research groups explored the use of metal halides—AlF_3_ and MgF_2_—as inorganic resists. Macaulay and colleagues, for example, applied a 16-nm-thick layer of amorphous aluminum fluoride (AlF_3_) on a thick carbon support via evaporation. The scholars then exposed the resist in STEM, utilizing 100-keV electrons and a high current density^37^. In Fig. 4, the SEM image displays impressive high-resolution results, featuring 7-nm-thick broad lines and 17-nm-wide spaces. Metal halides are self-developing resists that diverge from traditional e-beam resists, such as PMMA. When PMMA is exposed to an e-beam, chain breaking occurs. As a result, by immersing the sample in a liquid developer or heating it at a high temperature, the exposed PMMA areas can be removed. Conversely, metal diffusion and halide dissociation occur simultaneously when a metal halide is exposed to an e-beam, as shown in Fig. 5. Due to this unique property, no additional development is necessary after exposure.

**Fig. 6:**
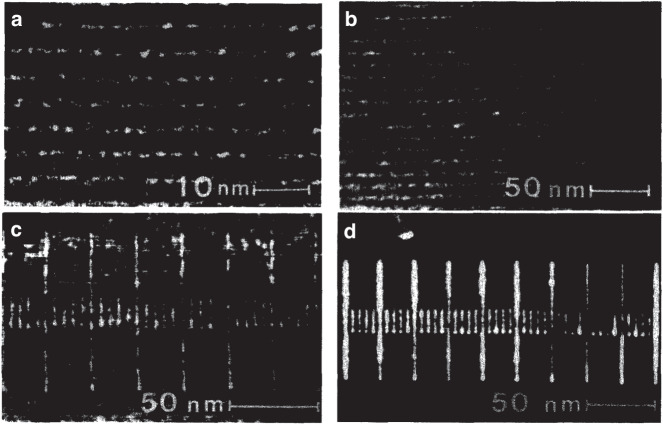
Patterns fabricated by using metal halides. **a** NaCl, **b** MgF_2_, **c** LiF, and **d** AlF_3_^38^. Reproduced with permission from AVS (1985)

**Fig. 7:**
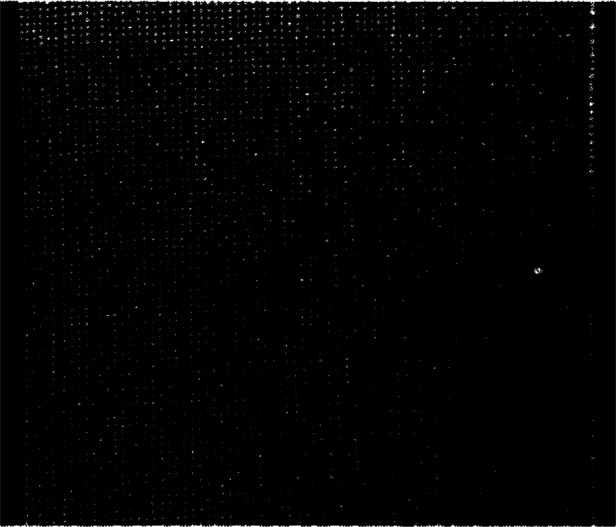
Hole array in an AlF_3_ film with a 2-nm diameter and 4-nm period^38^. Reproduced with permission from AVS (1985)

In addition to AlF_3_, several other materials were studied by Muray et al.^38^. In their study, these researchers exposed four metal halides—NaCl, MgF_2_, LiF, and AlF_3_—to a STEM system at 100 keV. While all materials demonstrated high resolutions (1–2 nm), the doses required for characteristic exposure varied significantly. LiF required a dose of 10^−2^ C/cm^2^, while 10^2^ C/cm^2^ was necessary to completely remove 50 nm of NaCl. AlF_3_ was deposited as an amorphous layer. Conversely, the other three materials formed polycrystalline films due to their relatively simple crystal structures and small unit cells. The authors noted that the amorphous nature of AlF_3_ led to more reproducible patterns than the other materials^38^. Figure 6 clearly shows that the patterns in the NaCl, LiF, and MgF_2_ films were noticeably less uniform than the AlF_3_ film. In contrast, Fig. 7 shows an ultrahigh-resolution hole array with a 2-nm diameter and 4-nm period composed of AlF_3_ and created by using a 1-nm diameter probe of 100-keV electrons with a 10 C/cm^2^ dose per hole. This procedure involved processing AlF_3_ films and using CHF_3_ plasma etching to transfer the pattern to the Si_3_N_4_ layer below. The selectivity between AlF_3_ and Si_3_N_4_ was 1:10 when the plasma power density was 0.15 W/cm^2^ at 30 mTorr. The pattern transfer was followed by removing the AlF_3_ using a 1:20 HCl solution.

Using evaporated metal halides in e-beam lithography allows for exceptional resolution and a user-friendly, development-free process. Nevertheless, it is important to note the limitations of this approach. These materials have low sensitivities, necessitating high doses and prolonged times for exposure, making them the most suitable for narrow trenches or hole arrays within a limited exposure area.

#### Evaporated polystyrene

In e-beam lithography, polystyrene (PS) is a popular negative resist^39,40^. PS can be dissolved in various solvents, including acetone, tetrahydrofuran, chlorobenzene, cyclohexane, xylene, and anisole. However, crosslinking occurs when PS is exposed to an electron beam, rendering these solvents ineffective^41,42^. PS resists have many benefits: for instance, low-molecular-weight PS has an exceptionally high resolution^41^, while high-molecular-weight PS has a high sensitivity^40^. In addition to being cost-effective, PS resists exhibit remarkably better resistance to plasma dry etching than PMMA^43^. Most importantly, PS can be evaporated, making it optimal for irregular surfaces where spin coating is not viable.

**Fig. 8:**
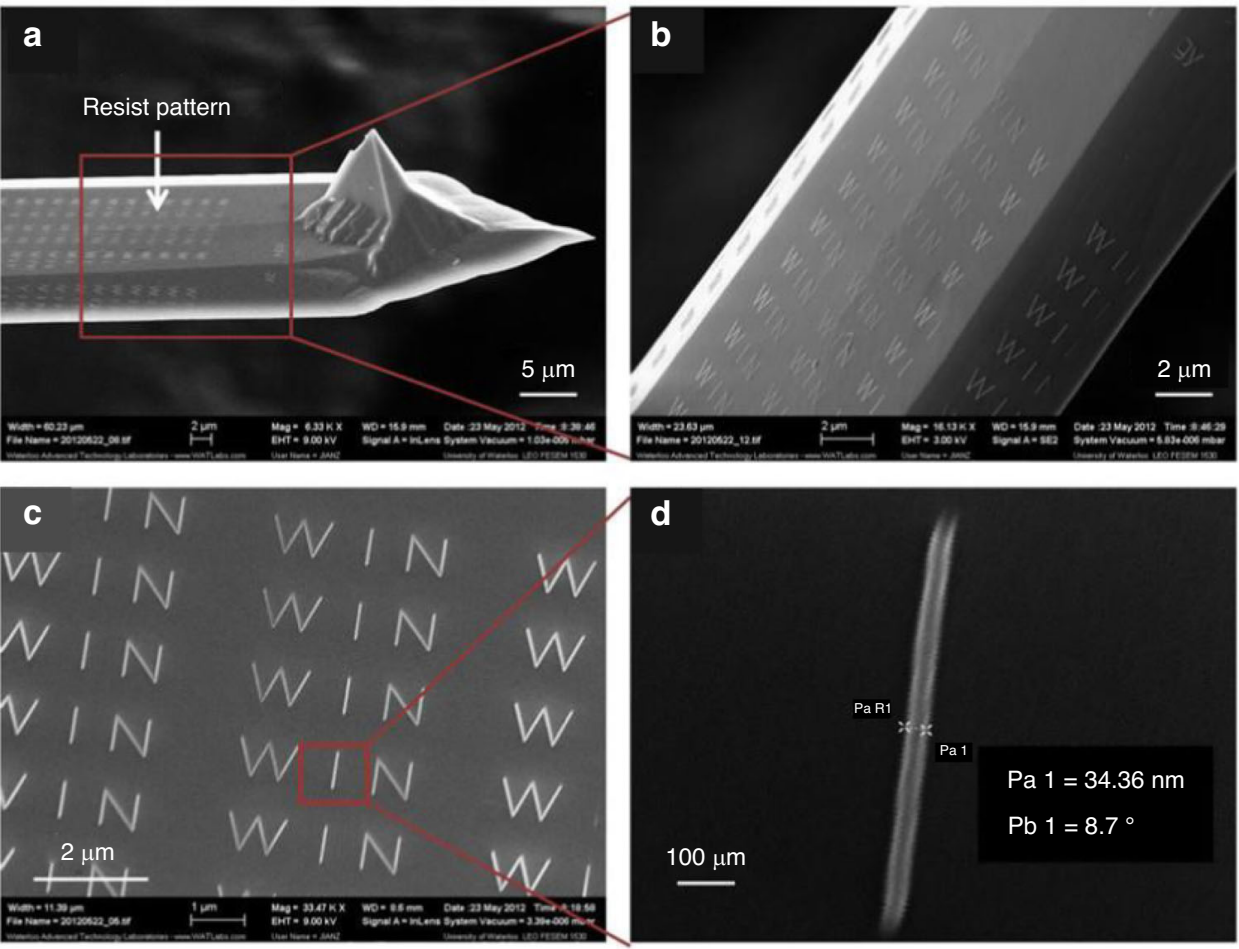
Example of evaporated PS patterned on an AFM cantilever. SEM images was taken at increasing magnifications from **a** to **d**^19^. Reproduced with permission from ACS Publications (2014)

**Fig. 9:**
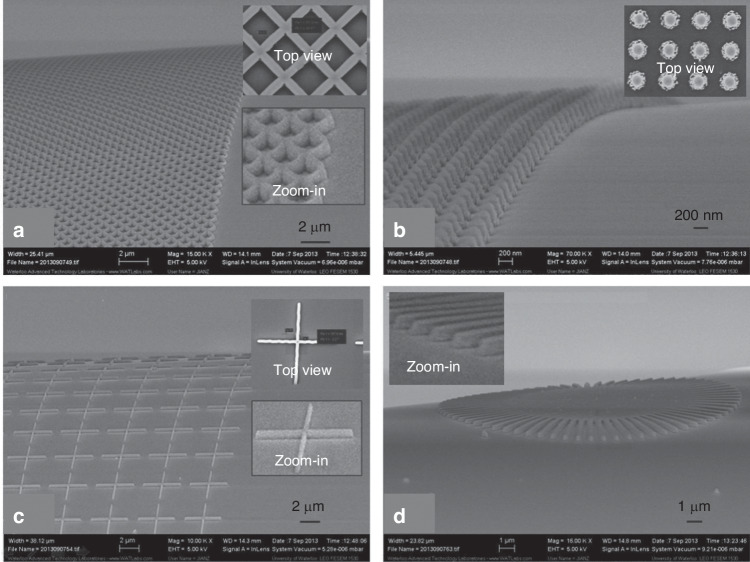
SEM images of nanostructures on an optical fiber with a height/depth of 270 nm. **a** 2D grating array with a line width of 167 nm; **b** dot array with a diameter of 200 nm; **c** crossbar array with a line width of 167 nm; and **d** star line pattern^19^. Reproduced with permission from ACS Publications (2014)

Considerable research has been conducted on e-beam lithography using evaporated PS in the last decade. Zhang et al.^19^ evaporated PS on an AFM cantilever that was a few micrometers wide and nonplanar. The researchers first evaporated low-molecular-weight PS (1.2 kg/mol) on AFM probes. Subsequently, the sample was exposed to a 5-keV e-beam and developed in xylene. Figure 8 shows SEM images of the PS “WIN” pattern on the cantilever, with a line width of 34 nm. Furthermore, Zhang et al.^19^ reported the use of evaporated PS in optical fiber fabrication, as shown in Fig. 9. The scholars began by depositing an Al sacrificial layer on a fiber surface (SiO_2_), followed by evaporating PS on the Al layer. After e-beam exposure and PS development, the pattern was transferred to Al and SiO_2_ with BCl_3_ plasma. Subsequently, CF_4_ plasma etching was performed.

**Fig. 10:**
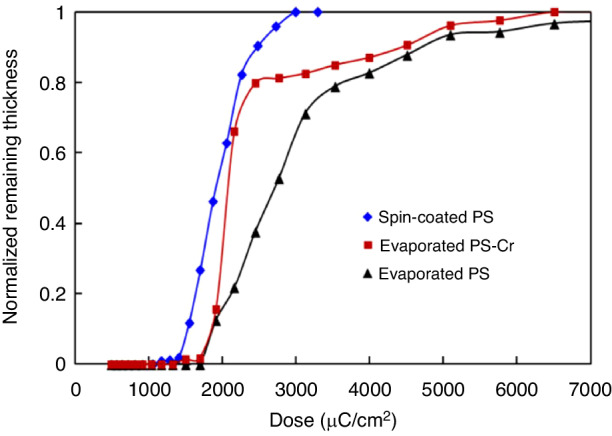
Contrast curves for spin-coated polystyrene (1.2 kg/mol), coevaporated polystyrene–Cr, and evaporated (pure) polystyrene (1.2 kg/mol). All resists were exposed to 5-keV e-beams and developed with xylene^20^. Reproduced with permission from IOP Publishing (2014)

**Fig. 11:**
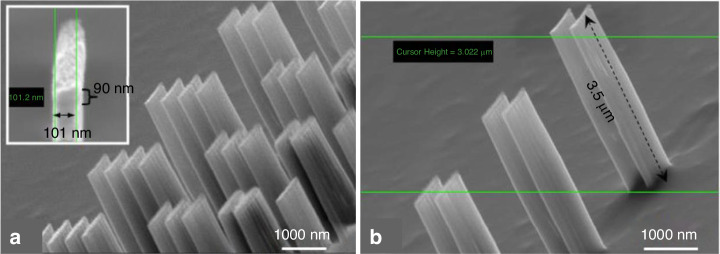
High-aspect-ratio silicon structures patterned by EBL using PS–Cr resist and ICP–RIE. **a** SEM image of silicon structures. The remaining PS–Cr mask is 90-nm thick, as shown in the inset. **b** A zoom-in view of the same structures. The structures are ~100 nm in width and 3.5 µm in height. Reproduced with permission from IOP Publishing (2014)

Reactive ion etching (RIE)^44,45^ is an effective method for etching high-aspect-ratio structures, but it requires a mask that can withstand the processing conditions until the desired height or depth is achieved. Unfortunately, common polymer resists are not suitable for plasma dry etching. Typically, the selectivity between silicon and PS is 1:1, which makes it difficult to etch deep patterns. To overcome this challenge, a hard mask composed of a metal or metal oxide is often used underneath the PS^46,47^. Metal usually has a selectivity reaching 1:100 (100 nm of Si is etched when 1 nm of metal is consumed). However, this approach is complicated and expensive. Con et al. recently discovered a new method for depositing nanocomposite masks by coevaporating Cr and PS^20^. The researchers first placed Cr and PS (1.2 kg/mol) in different crucibles in a thermal evaporator and then deposited a thin film composed of PS (20 nm) to ensure that Cr would not directly contact the substrate. The materials were then deposited simultaneously at a ratio of 1(Cr):15(PS). The total resist thickness was 200 nm. After deposition, the sample was subjected to e-beam exposure at 5 keV and soaked in xylene for 1 min. Contrast curves of three types of PS-containing resisted materials are shown in Fig. 10, which indicates that Cr did not dramatically affect PS properties at low concentrations. Then, the resistance of the Cr-PS resist was tested by etching with a nonswitching RIE process (22 sccm of SF_6_ and 38 sccm of C_4_F_8_, 10 mTorr of pressure, 1200 W of ICP power, 20 W of RF power, and 370 nm/min etching rate)^48,49^. The selectivity of the Cr-PS resist was 1:33, which was much greater than that of pure PS (1:2.6). Figure 11 shows the silicon structures with a height of 3.5 µm obtained by this method.

In summary, the process of resist evaporation is well developed and can be performed using conventional evaporation tools. This technique is highly productive and cost-effective since many samples can be mounted and coated with the resist in a single batch. However, it is crucial to acknowledge the potential drawbacks of evaporation. For instance, the shadowing effect may pose a challenge because the evaporated material may not be able to cover deep trenches or steep walls, thereby limiting its application on nonplanar surfaces. Additionally, positive resists, such as PMMA, cannot be evaporated due to their thermal decomposition process, which mainly involves unzipping.

### E-beam lithography using an ice resist

Over the last few decades, electron beam lithography using ice resists (iEBLs), also known as ice lithography, has gained the attention of researchers due to its high resolution in nanofabrication^50^. iEBL can easily achieve sub-10-nm-resolution features, even for multilayer 3D structures on irregular surfaces^22,51^. The specific properties of ice resists allow researchers to avoid spin coating and development and to integrate all the steps in a single instrument^52^. Water and organic solvents have been used to form ice resists. It has been recognized that water ice acts as a positive resist, while organic ice acts as a negative resist^23,50^.

#### Water ice resist

##### Process of water ice lithography

**Fig. 12:**
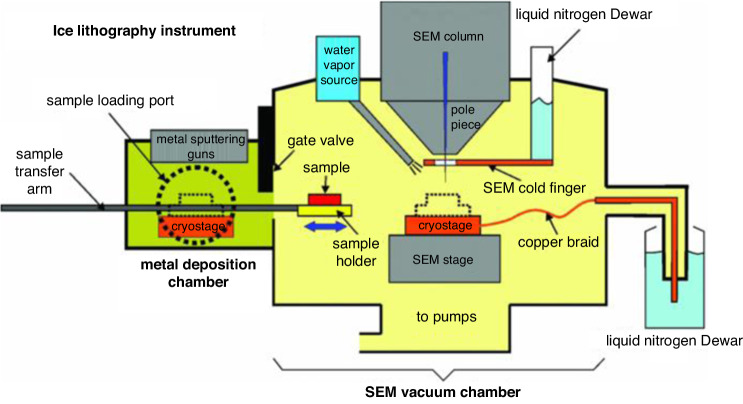
Ice lithography system^52^. Reproduced with permission from AIP Publishing (2011)

**Fig. 13:**
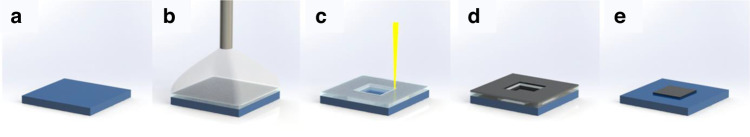
EBL process using water ice resists. **a** Refrigeration. **b** Water vapor injection and ice formation. **c** E-beam exposure. **d** Metallization. **e** Lift-off^51^. Reproduced with permission from ACS Publications (2018)

Due to the critical requirement of low temperatures to form ice, e-beam lithography instruments for water ice resists must be specifically designed. Han et al.^52^ reported the design of an ice lithography instrument built with a combination of commercial and custom-designed components (Fig. 12). This instrument contains four basic parts: a JEOL 7001 F scanning electron microscope (JEOL USA, Peabody, MA), a water vapor injector, a liquid nitrogen cooling system, and a metal sputtering chamber built in-house with a gate valve applied to isolate the two chambers. The SEM and metal sputtering chambers had a cryostage connected to an external liquid nitrogen dewar to keep the sample cold. The water vapor nozzle was mounted near the objective at an angle of 45 degrees to introduce water vapor during the e-beam exposure. To avoid the recondensation of ice removed by the e-beam, a large liquid nitrogen-cooled OFHC copper cold finger shield was applied directly above the cryostage. A schematic of the water ice lithography system is shown in Fig. 13^51^.

##### Fabrication of complex 3D structures by water ice lithography

**Fig. 14:**
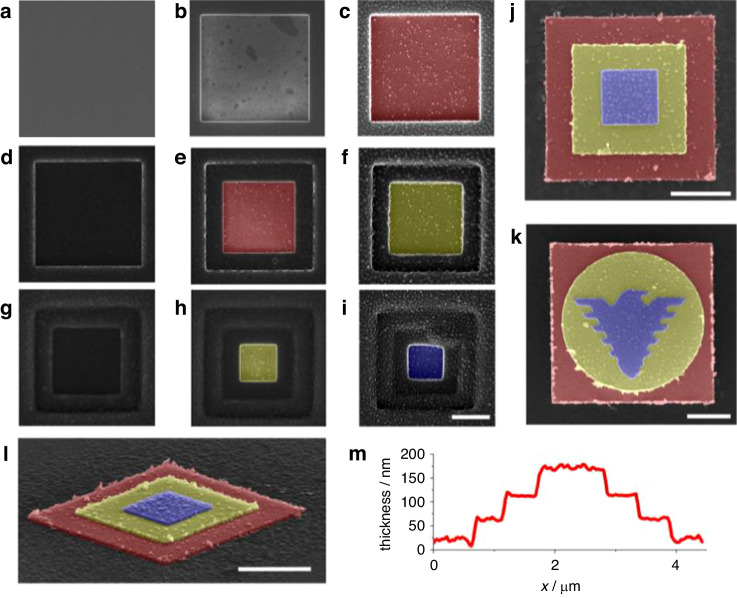
SEM images of 3D pyramidal structures fabricated by ice lithography. A 300-nm-thick ice resist was formed on sample surfaces **a**, **d**, and **g**. Squares (3 μm × 3 μm, 2 μm × 2 μm, and 1 μm × 1 μm) were formed on sample surfaces **b**, **e**, and **h** by a 20-keV/150-pA e-beam. The patterning dose was 0.8 C/cm^2^. Ag (60 nm) was deposited on sample surfaces **c**, **f** and **i**. SEM images of the final pattern after lift-off **j** and **l**. Another sample with a different pattern was created via the same process **k**. Central line scan of the 3D pyramidal nanostructure **l** by atomic force microscopy (AFM) (**m**)^51^. Reproduced with permission from ACS Publications (2018)

The e-beam lithography process involves several steps—spin coating, e-beam exposure, and resist dissolution in liquid solvents^53^—each requiring different instruments. Fabrication of complex 3D structures, such as the stacked layered structures shown in Fig. 14, can be very difficult and time-consuming^51^. In ice lithography, the entire process is greatly simplified since the resist coating system (water vapor nozzle) and metal deposition system are combined with the exposure system, indicating that the sample is never removed from the instrument. Moreover, e-beams in ice lithography are applied to vaporize ice. Thus, development occurs simultaneously with exposure, making this procedure a self-developing process. However, several problems have been observed in ice lithography. Hong et al.^51^ reported that the ice layer sublimates and recrystallizes on the sample surface, leading to uneven metal layers when the sample temperature is not maintained at a sufficiently cold level. This problem may be solved by switching to an improved cooling system^50^. Another issue was identified by Han et al.^22^, who reported that metal structures created by ice lithography have poor electrical qualities because metal films deposited on cold surfaces tend to be nanoporous^54^. However, the scholars found that annealing samples at temperatures ranging from 300 to 600 °C in an Ar atmosphere could significantly improve the electrical performance.

##### Water ice lithography on irregular surfaces

**Fig. 15:**
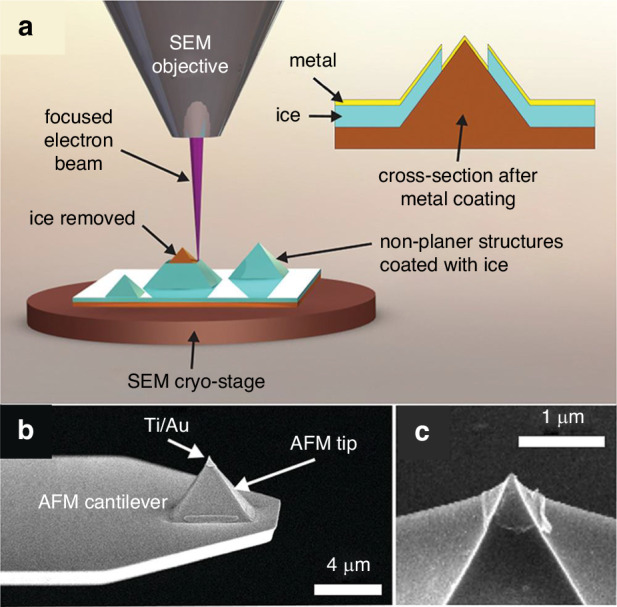
Ice lithography on a commercial AFM tip. Process of ice lithography and Au deposition **a**. SEM images of the final results **b**, **c**. The ice resist was 400-nm thick. An e-beam dose of 3 C/cm^2^ with 100 pA at 15 keV was used to pattern the rectangle on the AFM tip^55^. Reproduced with permission from ACS Publications (2012)

Ice lithography is a good choice for fabricating irregular surfaces, such as AFM tips. Han et al.^55^ fabricated a small metallic cap on the apex of a tip using an SEM technique specifically modified for ice lithography^52^. Figure 15 shows their detailed process. The scholars first loaded the sample onto a cryostage and cooled it to <120 K. Water vapor was injected, and ice formed, covering the entire tip surface. A focused e-beam was used to remove the 600 nm × 600 nm square pattern. Without breaking the vacuum, the AFM tip was moved to the deposition chamber, where 1 nm of Ti and 20 nm of Au were coated (Fig. 15a). Finally, the lift-off process was performed by immersing the sample in isopropyl at room temperature. The results for the metal-coated AFM tip are shown in Fig. 15b, c. The Ti and Au caps can be clearly observed. Patterning AFM tip apexes has many applications, such as tip-enhanced Raman spectroscopy^1^, magnetic force microscopy, and scanning single-electron transistor microscopy^3^. After fabricating a small metal cap (usually composed of Cr or Al), the tip can be etched by reactive ion etching (RIE) to increase the aspect ratio of the apex, significantly minimizing tip artifacts^56^.

**Fig. 16:**
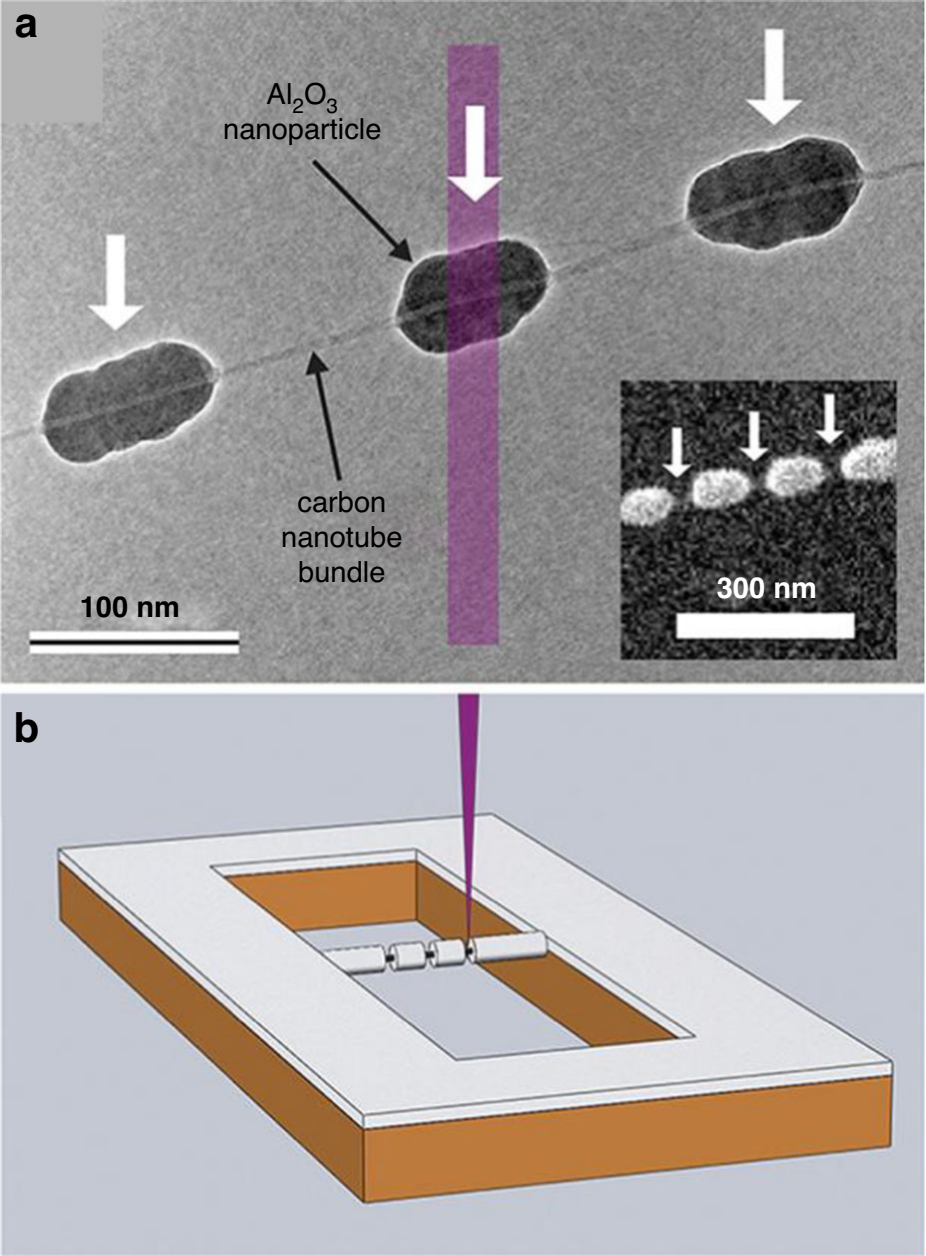
Ice lithography on carbon nanotubes. **a** TEM image of Al_2_O_3_ nanoparticles grown by ALD on a bundle of SWCNTs on which 5 Å of Ti had previously been deposited during ice lithography. The white arrows show the locations where ice was removed. **b** Schematic of the experiment (not to scale). An e-beam dose of 3 C/cm^2^ (100 pA at 30 keV) was used to pattern a 60-nm-thick layer of ice^55^. Reproduced with permission from ACS Publications (2012)

Single-walled carbon nanotubes (SWCNTs) have been studied extensively by many researchers. Normal lithography by spin coating on SWCNTs does not work since they are too fragile and nonplanar. Han et al.^55^ demonstrated a method for patterning SWCNTs by ice lithography (Fig. 16). The SWCNTs were grown in 1-μm trenches of a free-standing Si_3_N_4_ membrane. Ice was formed to cover the entire sample surface. Several 25-nm-long regions were then removed (white arrows in Fig. 16). After withdrawing the sample to a deposition chamber, 5 Å of Ti was sputtered to form an adhesive coating on the ice-free regions. Lift-off was performed using isopropyl alcohol at room temperature. Then, atomic layer deposition (ALD) of Al_2_O_3_ was conducted. When the sample was exposed to air, a surface oxide of Ti was formed, which initiated the ALD process since it could not begin on pristine SWCNTs^57^.

#### Organic ice resist

##### Process of organic ice lithography

Similar to water ice lithography, the instrument for organic ice is specifically designed for gas injection and maintaining low temperatures. Figure 18 shows the design of an organic ice lithography instrument made by Tiddi et al.^58^. There were four basic components of this instrument. The most important component was an LEO scanning electron microscope (SEM) from Zeiss with an e-beam lithography system (ELPHY Quantum from Raith Gmbh, Germany). A sample load lock was mounted and connected to the SEM chamber to quickly change the sample and sublimation. The other two components were gas injection and liquid nitrogen cooling systems, which cooled the sample and allowed the formation of organic ice layers. Figure 19 shows the lithography process. The sample was loaded from the load lock and mounted onto the cryostage in the SEM chamber. Then, the sample was cooled to 120–150 K^23^, after which organic gas injection was performed to form an ice layer on the sample surface. After ice resistance coating, an e-beam was applied to expose the desired areas. Instead of being vaporized, crosslinking occurred in organic ice when it was exposed to an e-beam. Thus, organic ice served as a negative resist. Finally, the sample was transferred to the load lock and heated to room temperature. With the sublimation of unexposed organic ice, exposed and crosslinked areas remained. Many organic chemicals could be used for ice lithography, such as C_5_H_12_O, C_7_H_8_O, C_8_H_18_, C_9_H_20_, C_11_H_24_, C_14_H_30,_ and C_3_H_8_O^23,59,60^.

##### Organic ice lithography on irregular surfaces

**Fig. 17:**
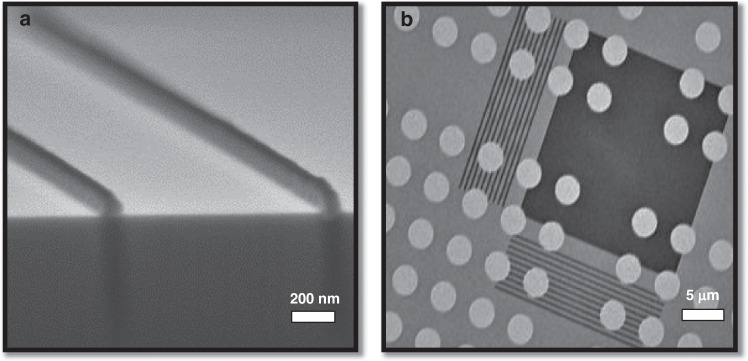
Fabrication of patterns on an irregular substrate. **a** SEM image of 60-nm-thick anisole ice lines on the very edge of a chip. **b** TEM image of lines and squares on a porous membrane^23^. Reproduced with permission from ACS Publications (2017)

Patterning irregular surfaces is achievable by organic ice lithography. Tiddi et al.^23^ obtained 60-nm-wide anisole ice lines, which reached the very edge of the sample (Fig. 17a). The figures show the lines and squares on the porous Si_3_N_4_ membrane (Fig. 17b). All the lines and squares had well-defined shapes and were not influenced by the previous surface structures.

Unlike water ice, which cannot exist under ambient conditions and needs low temperatures to be maintained during processing, organic ice is very stable at room temperature and even under dry etching conditions^22,61^. These characteristics indicate that organic ice can be used as a reactive ion etching (RIE) mask. After dry etching, the remaining organic ice resist can be easily removed by O_2_ plasma.

Overall, water and organic ice lithographic processes dramatically increase the efficiency of EBL with only a single loading–unloading cycle, regardless of the number or complexity of the patterned layers. These approaches minimize contamination on the sample surface because of their single operation, clean development, and lift-off without the use of aggressive solvents. Moreover, these techniques have strong potential for patterning irregular and fragile substrates, suggesting that they will play an important role in future research. However, the industrialization of these processes poses a challenge due to the requirement for a specifically designed tool. Moreover, the types of ice utilized in these procedures are extremely unresponsive, restricting the amount of exposed area.

### E-beam lithography using a grafted monolayer polymer brush

Although it is possible to apply ice resists or evaporate resists to nonplanar surfaces, these resists all suffer from very low sensitivities and high costs^24^. There is still a great demand for a good resist that is low cost and can be easily coated on irregular and nonplanar surfaces for high-resolution patterning. A monolayer polymer brush is a good choice for achieving this goal. PMMA (polymethyl methacrylate) and PS (polystyrene) are two commonly used materials for this purpose. This method has a variety of advantages. PMMA and PS are popular and inexpensive polymers that can be coated onto a substrate by spin or dip coating^25^. In addition, the monolayer is chemically/firmly bonded to the substrate. Therefore, there is no pattern collapse because of capillary force when developing in a liquid^62^. Moreover, this process eliminates the edge-bead effect^63^; thus, it can be used to pattern extremely small substrates.

#### Grafted PMMA brush

##### PMMA brush as a positive resist

**Fig. 18:**
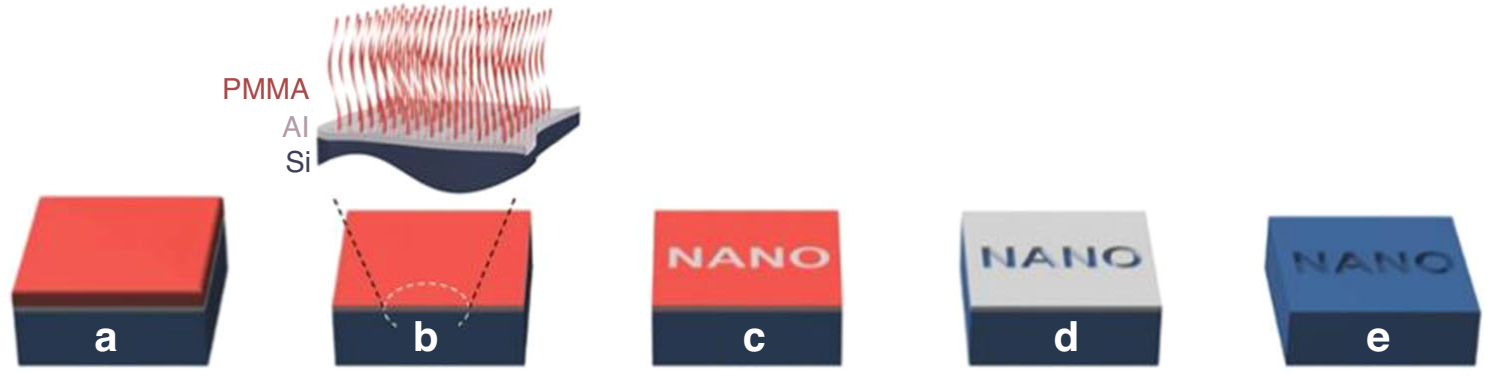
Schematic diagram of the fabrication process. **a** First, the Al is first deposited; then, the PMMA film is coated; and finally, annealing is performed. **b** The bulk PMMA was washed away, leaving behind a monolayer brush. **c** The e-beam was exposed and developed. **d** Wet-etched Al. **e** Dry-etched substrate^25^. Reproduced with permission from Wiley-VCH (2016)

PMMA is a classic positive resist for e-beam lithography. To form a monolayer brush, Dey et al.^25^ chose PMMA with 1.6% MAA (methacrylic acid) monomer; this material behaves similarly to pure PMMA. PMMA samples with MAA contents reaching 40% can still act as positive resists in e-beam lithography^64^. The process starts with depositing a hard mask, spin coating PMMA, dissolving the polymer chains, exposing the sample, developing the sample, wet etching the hard mask, and drying the substrate (Fig. 18).

PMMA contains 1.6% MAA, which has a carboxyl (-COOH) group and can further promote the grafting process with materials terminated with a hydroxyl (-OH) group^65^. The -COOH) group of P(MMA-co-MAA) can react with Si substrates. The same type of chemical bond can be found between PMMA and Al since the -OH group exists in the native oxide layer of Al^66^. Because the monolayer PMMA brush was too thin to transfer the pattern directly, Dey et al. deposited a hard mask layer (8–10 nm Al) on the substrate. After Al deposition, PMMA was spin-coated on the Al layer, and the -COOH and -OH groups reacted during annealing at 160 °C for 24 h. The bulk PMMA was then removed by washing with acetic acid for 1 min^26^. Only a 9-nm-thick monolayer PMMA brush remained due to the firm bonding with the Al layer.

**Fig. 19:**
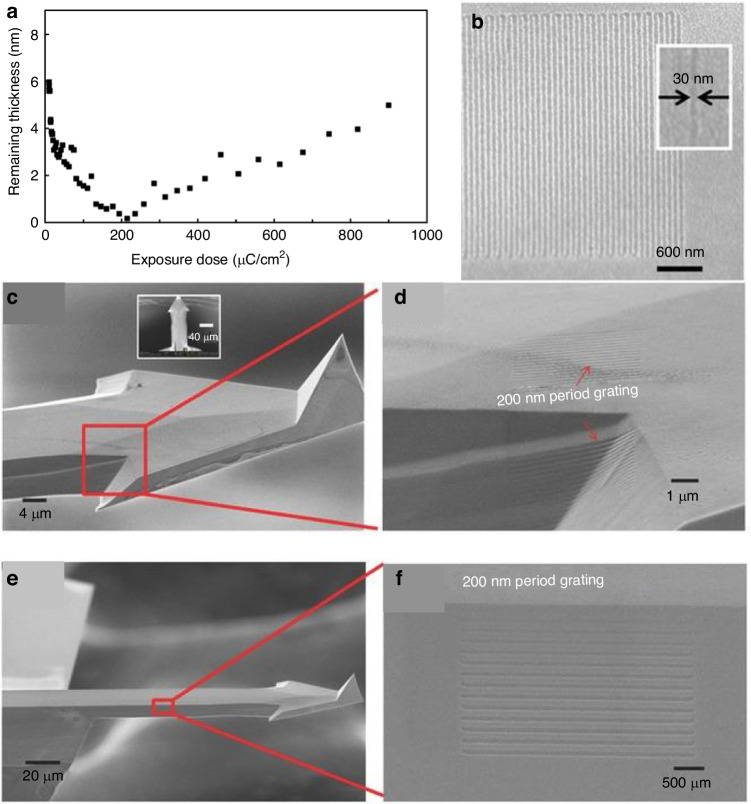
EBL on an AFM cantilever by using PMMA brush. **a** Contrast curve of a 9-nm-thick monolayer PMMA brush (1.6% MAA). **b** SEM image of the grating pattern obtained using a monolayer PMMA brush followed by pattern transfer, showing high-resolution lines with a width of 30 nm. **c**, **d** SEM images of a grating fabricated near the corner exposed at 66 pC/cm. **e**, **f** SEM images of the grating on the side slope at 56 pC/cm^25^. Reproduced with permission from Wiley-VCH (2016)

A thick PMMA layer is positive at a low dose and negative at a very high dose since both chain crosslinking and scission occur; crosslinking dominates at a high dose^67,68^. The monolayer PMMA brush exhibits the same properties (Fig. 19a). The contrast curve confirms that the monolayer brush behaves as a positive resist in low-dose areas and then gradually changes to a negative resist when the dose exceeds 200 μC/cm^2^ at 3 keV. Similar to thick PMMA, developer MIBK (methyl isobutyl ketone):IPA (2-propanol) is still effective for monolayer brushes. Interestingly, acetic acid, which is a good solvent for PMMA, can be used to develop a monolayer brush. Acetic acid will destroy the entire structure of thick PMMA since monolayer PMMA is strongly bonded with the sublayer and cannot be dissolved by acetic acid.

After development, the whole pattern can be transferred to Al by wet etching and then to the Si substrate by dry etching. A high-resolution 30-nm-thick line can be obtained by this method (Fig. 19b). Moreover, the monolayer PMMA brush performs well for nonplanar substrates (Fig. 19c–f).

##### PMMA brushes as negative resists

**Fig. 20:**
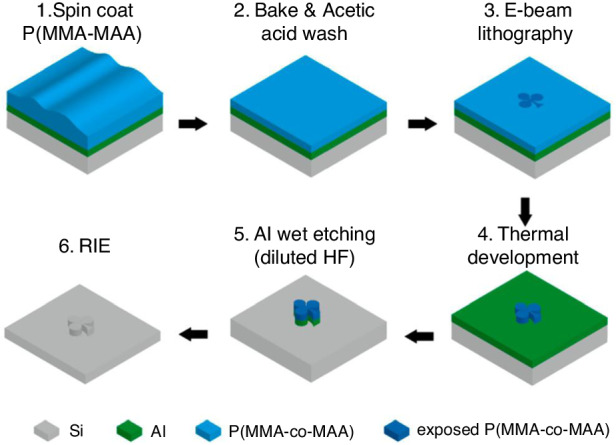
Schematic diagram of the fabrication process^26^. 1. Spin coat PMMA (1.6% MMA). 2. Wash away bulk PMMA. 3. E-beam lithography. 4. Thermal development. 5. Etch Al in diluted HF. 6. Transfer the pattern into silicon by RIE. Reproduced with permission from ACS Publications (2017)

PMMA is known to become a negative resist at very high doses. However, typical developers (MIBK:IPA) are not applicable to monolayer PMMA brushes due to the chemical bonding of PMMA molecules on the surface. In this case, thermal development is used to remove unexposed PMMA brushes. Yamada et al.^26^ reported this process, as shown in Fig. 20, employing a monolayer PMMA (1.6% MMA monomer) brush as the negative resist for sample fabrication.

*Thermal development and Al wet etching*

**Fig. 21:**
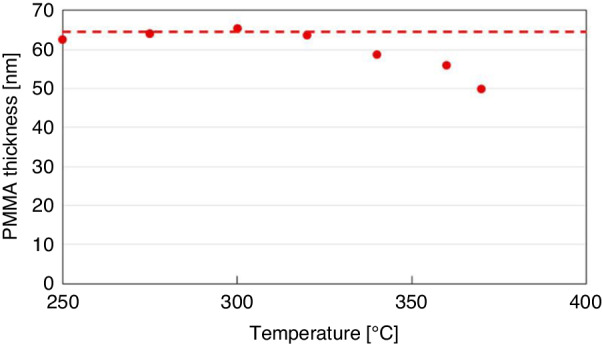
Thickness of PMMA after 1 min of thermal treatment. The dashed line shows the PMMA thickness before thermal treatment^26^. Reproduced with permission from ACS Publications (2017)

**Fig. 22:**
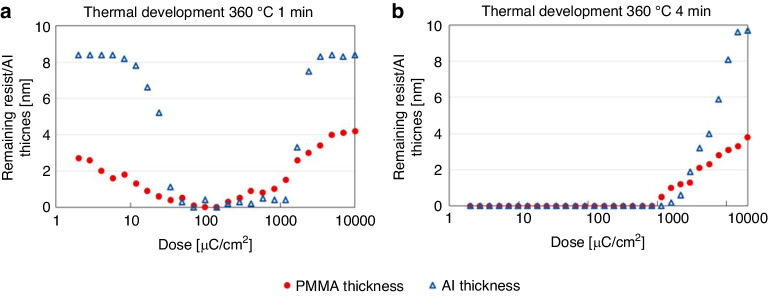
Thicknesses of PMMA after development and the Al layer after Al wet etching. The conditions were thermal development at 360 °C for (a) 1 min and (b) 4 min. Al wet etching was performed by soaking the film in diluted HF for 11 s^26^. Reproduced with permission from ACS Publications (2017)

Figure 21 shows the evaporation rate of PMMA after 1 min of thermal treatment on a hotplate. The PMMA layer started to evaporate when the temperature reached ~320 °C. To obtain a relatively high evaporation rate, a temperature of 360 °C was chosen. After thermal development, the pattern of the PMMA layer was transferred to an Al layer by soaking the sample in diluted HF solution (HF:DIW 1:250 dilution ratio) for 11 s. After a 1 min of thermal treatment, the PMMA layer persisted in unexposed and heavily exposed areas, providing good protection for the Al layer in HF wet etching (Fig. 22a). However, the treatment duration was insufficient, and incomplete negative behavior of monolayer PMMA remained. The thermal development time was increased to 4 min to improve the performance, and only portions of the PMMA layer with very high doses remained (Fig. 22b).

*Fabrication on planar and nonplanar silicon surfaces*

**Fig. 23:**
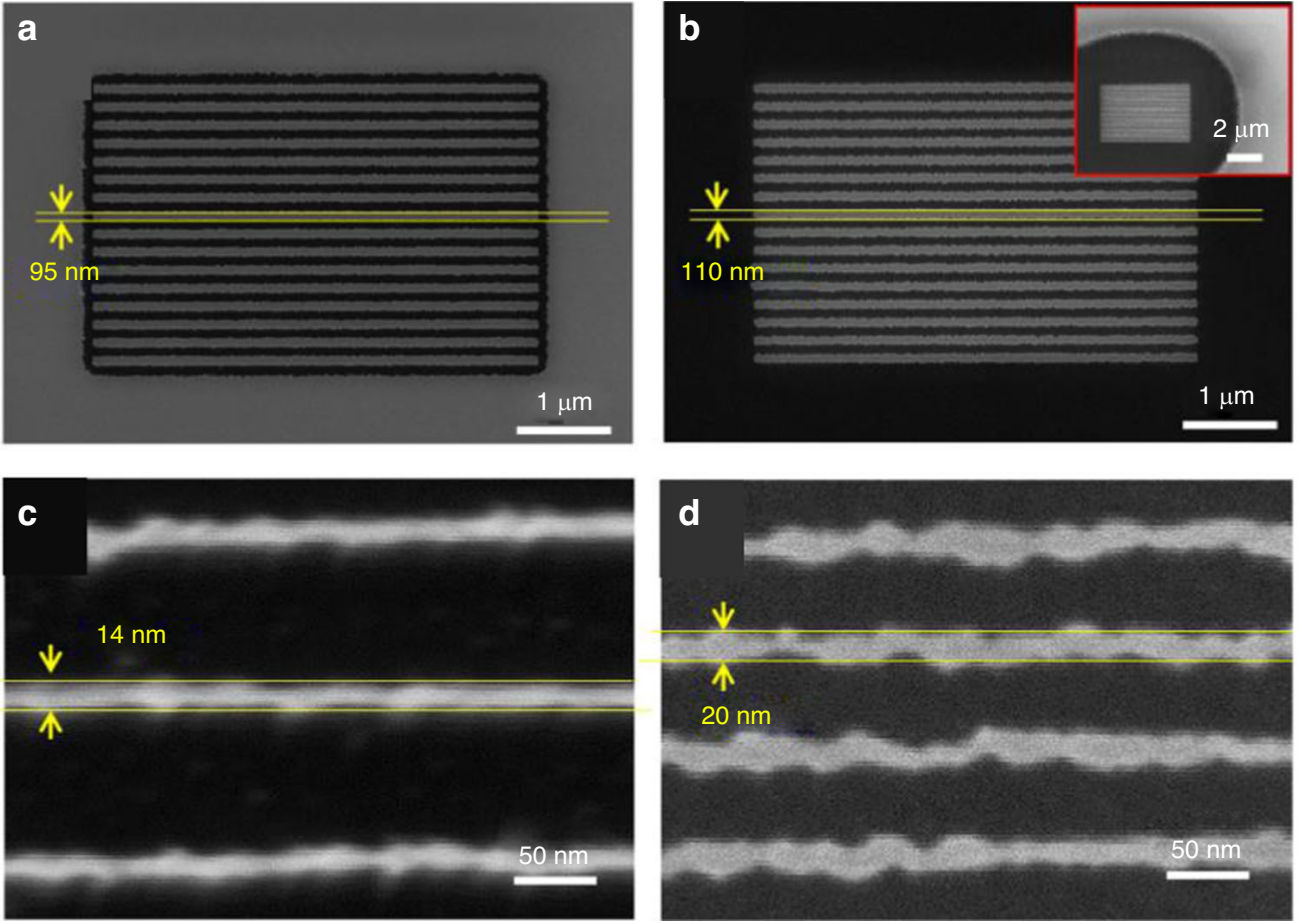
SEM images of line arrays etched into silicon. The electron energy and dose are **a** energy of 3 keV, area dose of 3000 μC/cm^2^, width of 100 nm, pitch of 200 nm; **b** energy of 20 keV, area dose of 33,000 μC/cm^2^, width of 100 nm, pitch of 200 nm (the inset figure shows the same pattern at a relatively low magnification); **c** energy of 3 keV, line dose of 20 nC/cm, pitch of 100 nm; and **d** energy of 20 keV, line dose of 120 nC/cm, pitch of 60 nm^26^. Reproduced with permission from ACS Publications (2017)

**Fig. 24:**
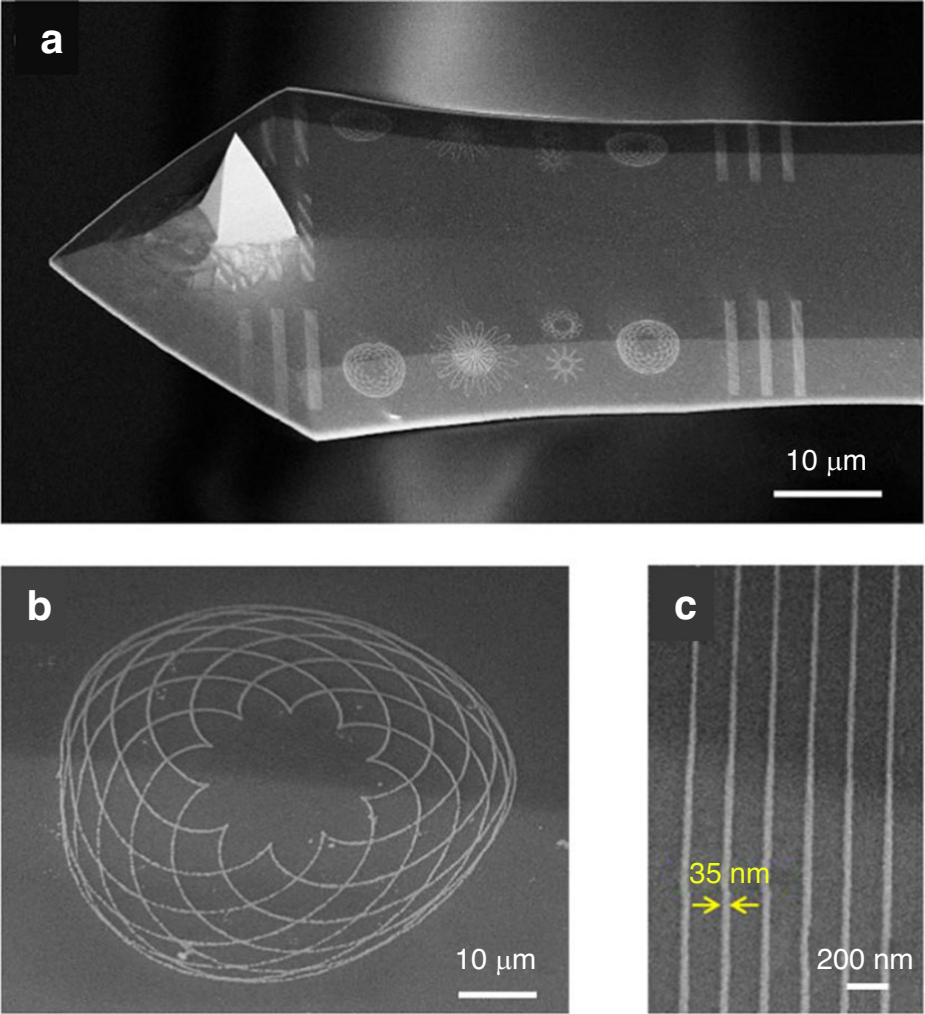
SEM images of line and geometrical patterns fabricated on a nonplanar region (edge) of an AFM cantilever. **a** Top view; **b** magnified image of a cycloid pattern exposed at 20 keV and 400 nC/cm; and **c** magnified image of a line pattern exposed at 20 keV and 400 nC/cm with a period of 200 nm^26^. Reproduced with permission from ACS Publications (2017)

Patterns can be transferred into silicon by fluorine-based plasma etching. A high-resolution line with a width of 14 nm was achieved using this method (Fig. 23c). In addition, Fig. 24 shows several intricate geometrical patterns, including lines and cycloids, on the nonplanar surface of an AFM cantilever.

#### Grafted PS (Polystyrene) brushes

**Fig. 25:**
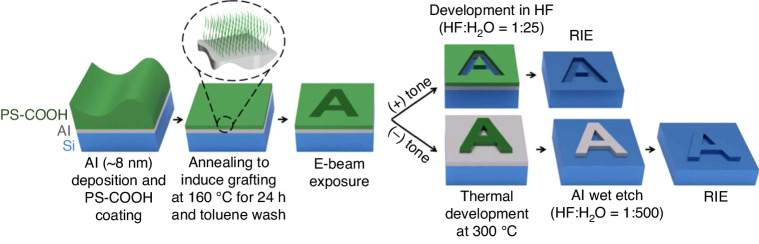
Process steps for patterning substrates using a PS brush as a monolayer resist for both negative and positive tones^69^. Reproduced with permission from ACS Publications (2017)

The process of performing e-beam lithography using a monolayer PS brush is very similar to that using a PMMA brush developed by Aydinoglu et al.^69^, as shown in Fig. 25. PS is spin-coated on the Al hard mask layer, and only a single layer of PS remains after washing with toluene. PS is naturally a negative resist because an electron beam induces crosslinking, and thick PS layers can be developed by common solvent developers^41,70^. However, solvent developers are insufficient in the case of monolayer PS because of the strong bond between the -COOH groups in the PS layer and the -OH groups in the Al layer. To solve this problem, thermal development can be used in place of solvent. Moreover, monolayer PS can serve as a positive resist when using an unconventional development method, where HF solution is used to etch the Al layer underneath, as shown in Fig. 25.

##### PS brushes as negative resists

**Fig. 26:**
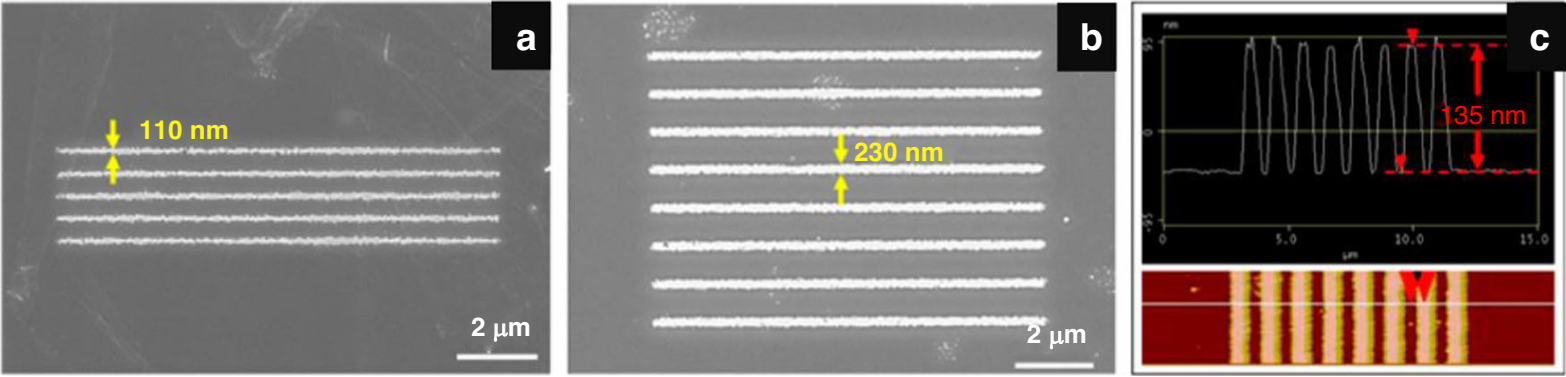
SEM images of the line array pattern obtained using a PS brush as a negative resist. **a** 500-nm period, 2.2-nC/cm exposure dose, **b** 1-μm period, 3.8-nC/cm exposure dose, and **c** AFM image of the line array presented in **b**^69^. Reproduced with permission from ACS Publications (2017)

Monolayer PS brushes can be thermally developed since exposed and crosslinked PS has greater thermal stability than unexposed linear PS^71^. However, if the temperature is too high ( > 350 °C), all the PS will evaporate. Conversely, if the temperature is too low ( < 250 °C), the unexposed area is not fully vaporized. Aydinoglu et al.^69^ optimized the process to 300 °C for 1 min. After development, the pattern was transferred to the Al layer using a very dilute HF solution (1:500). Otherwise, the thin PS brush could not adequately protect the Al sublayer. RIE dry etching was then performed to transfer the pattern to the substrate. Figure 26 shows the results obtained using a PS brush as a negative resist for 110-nm-wide lines. Here, the resolution was limited by the random nature of PS brush vaporization (desorption) and the proximity effect^69^.

##### PS brush as a positive resist

**Fig. 27:**
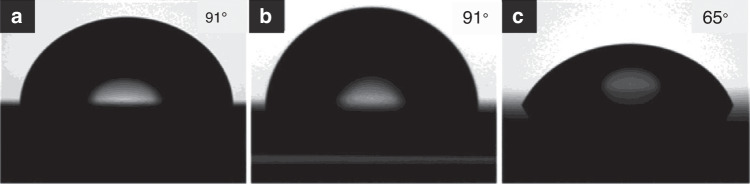
Contact angle measurements. **a** a thick PS film, **b** a noncrosslinked PS brush, and **c** a crosslinked PS brush^69^. Reproduced with permission from ACS Publications (2017)

An unconventional development method can be conducted to create a positive resist from a monolayer PS brush. PS is a renowned negative e-beam resist that can be crosslinked via electron exposure. However, the PS brush and the underlying Al layer can act as a positive resist. After lithography, if the sample is directly soaked in a diluted HF (1:25) solution for 15 s without any thermal treatment, HF will penetrate the crosslinked PS and etch the Al layer underneath while maintaining the unexposed area. This phenomenon is possible because the exposed and crosslinked PS becomes much more hydrophilic than the unexposed PS. The contact angle measurements (Fig. 27) indicate that the hydrophilicity changes after electron beam exposure, with the contact angle decreasing dramatically from 91° to 65°^69^.

**Fig. 28:**
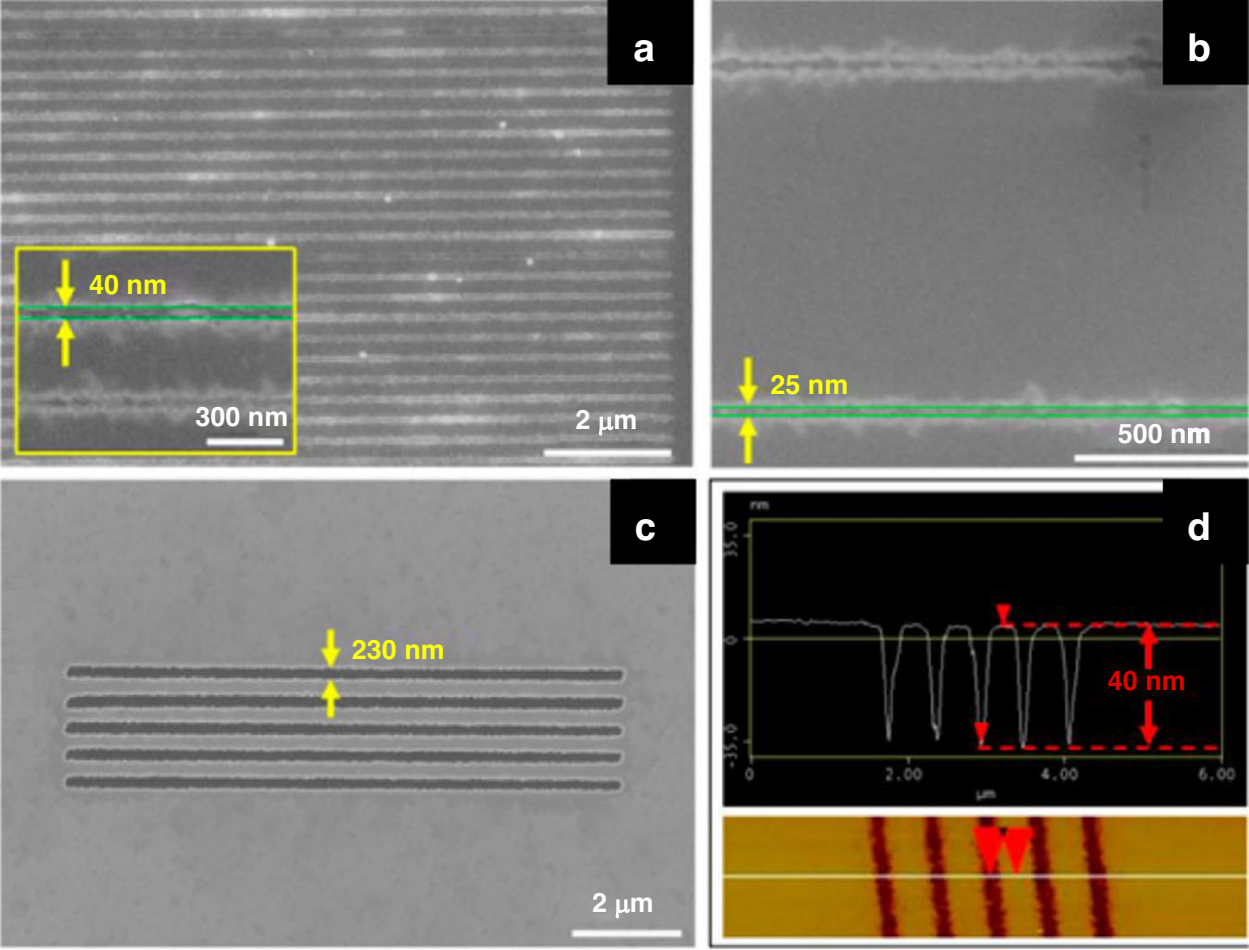
SEM images of the pattern obtained by using a PS brush as a positive resist. **a** 300-nm period, 0.2-nC/cm exposure dose; **b** 1-μm period, 0.2-nC/cm exposure dose; **c** 500-nm period, 3.8-nC/cm exposure dose; and **d** AFM image of the line array presented in **c**^69^. Reproduced with permission from ACS Publications (2017)

**Fig. 29:**
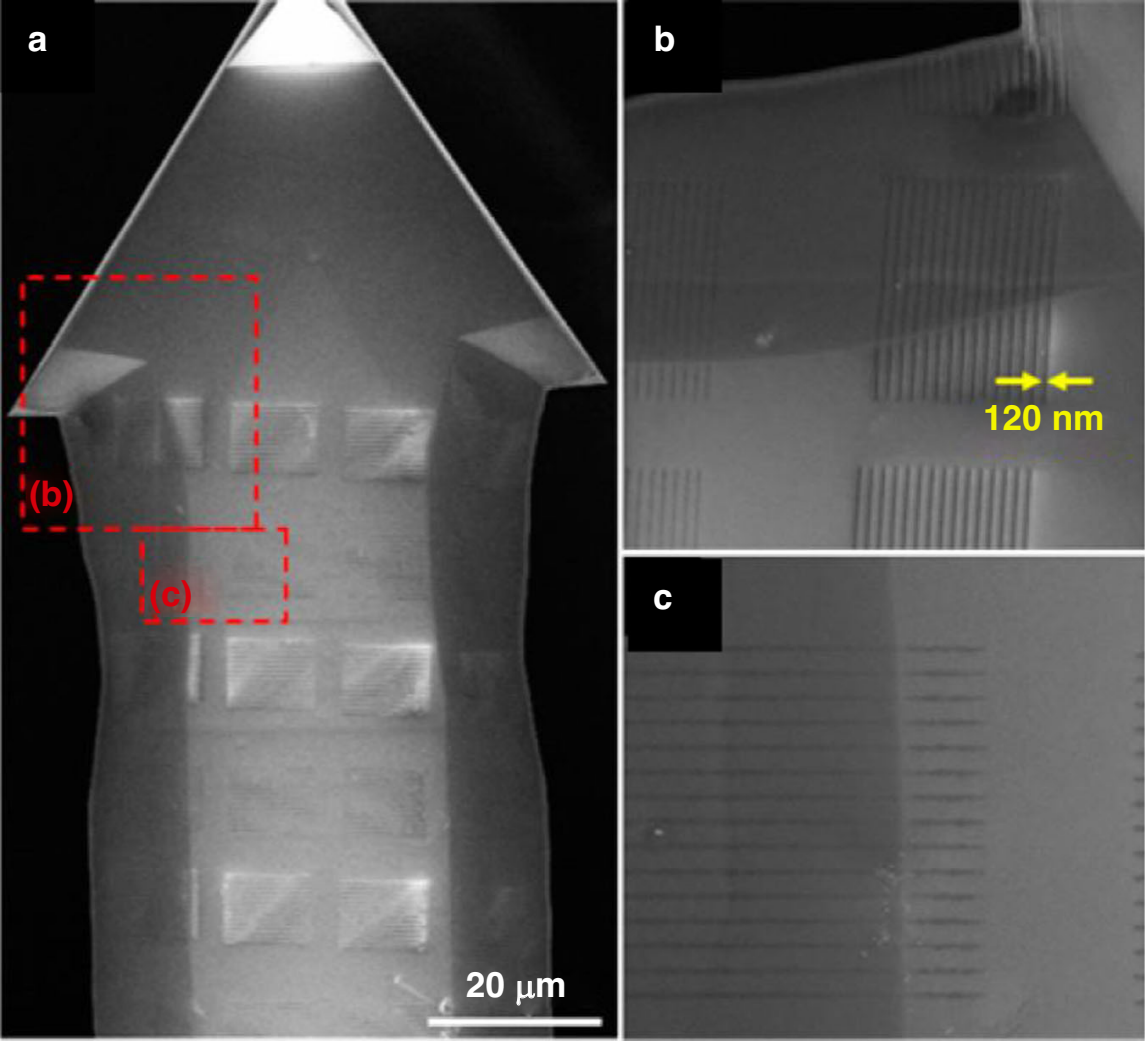
SEM images of the AFM probe grating patterns. **a** top view, **b** magnified view: 500-nm period, 2.6-nC/cm dose, and **c** 500-nm period, 1.2-nC/cm dose^69^. Reproduced with permission from ACS Publications (2017)

Lines with widths of 25 nm can be obtained using monolayer PS as a positive resist (Fig. 28). The resolution is much greater than that when PS is used as a negative resist. Figure 29 shows that monolayer PS can be fabricated on nonplanar surfaces such as AFM cantilevers with high resolution.

In summary, the use of grafted PMMA and PS provides a cost-effective and straightforward approach for uniformly coating resists onto irregular surfaces. This technique is easy to apply in the current industry because no special materials or tools are used. This process chemically bonds a monolayer to the sample surface, preventing pattern collapse due to capillary forces during development and edge beads when utilizing relatively thick resists. However, spin or dip coating is used in this method, suggesting that surfaces with significant depth variation may not be suitable.

### E-beam lithography using a self-assembled monolayer (SAM)

Self-assembled monolayers (SAMs) begin to form when molecules from vapor or liquid interact and strongly connect with the substrate surface via head groups^72–75^, typically thiols, silanes, and phosphonates. Chemical bonds are formed between the head groups and the substrate. This monolayer can be used as an e-beam resist to fabricate microstructures and nanostructures. Different chemical reactions are used to create SAMs depending on the substrate material. Thiols (-SH) bind to Au, while Ag, Cu, and hydroxy (-OH) groups bind to Si^73^.

#### SAM as an e-beam resist on metals

**Fig. 30:**
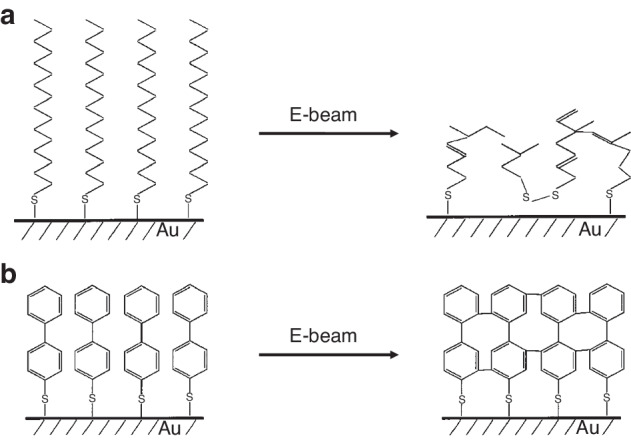
Reactions of SAMs under e-beam exposure. **a** positive aliphatic SAMs. Chain cleavage happens under exposure. **b** negative aromatic SAMs. Crosslinking occurs under exposure^80^. Reproduced with permission from Elsevier (2012)

**Fig. 31:**
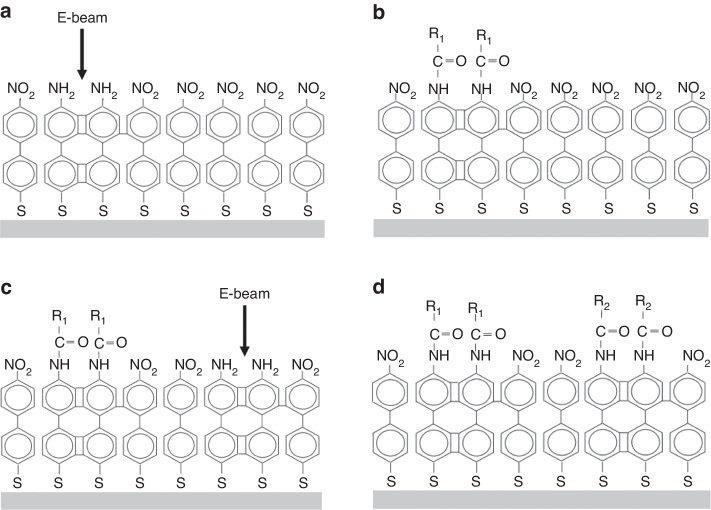
Schematic of electron-induced chemical lithography. **a** An electron beam converts the terminal nitro groups of a 4’-nitro-1,1’-biphenyl-4-thiol monolayer to amino groups while the underlying aromatic layer is crosslinked. **b** The crosslinked aminobiphenyl thiol region is used for selectively coupling molecules (R1). **c**, **d** The procedure can be repeated to immobilize additional molecules (R2) on the same surface^79^. Reproduced with permission from Wiley-VCH (2001)

Thiols (-SH) are widely used to connect gold and SAMs. Moreover, research has shown that other metals, such as silver and copper, can also form SAMs via -SH groups^76–78^. The reaction between a metal surface and molecules containing thiols is as follows:

$${{\rm{X}}({{\rm{CH}}}_{2})}_{{\rm{n}}}{\rm{SH}}+{{\rm{Au}}}^{0}\to {{\rm{X}}({{\rm{CH}}}_{2})}_{{\rm{n}}}{{\rm{S}}}^{-}{{\rm{Au}}}^{{\rm{I}}}+1/2{{\rm{H}}}_{2}$$

where X is a tail group. Several SAMs with different molecular structures can be used as e-beam resists. Researchers have manufactured both positive and negative resists with SAMs. Figure 30 shows two types of SAM resists. In aliphatic SAMs, electrons induce the cleavage of C–H bonds to form C=C double bonds, thus resulting in a positive resist. The C–H bonds cleave in aromatic SAMs, followed by crosslinking between neighboring phenyl units, resulting in a negative resist. Similarly, C–H cleavage and crosslinking occur in aromatic SAMs terminated with nitro groups. However, liberated H atoms locally reduce -NO_2_ to -NH_2_ groups, which can be further chemically modified by electrophilic agents (Fig. 31)^79,80^.

**Fig. 32:**
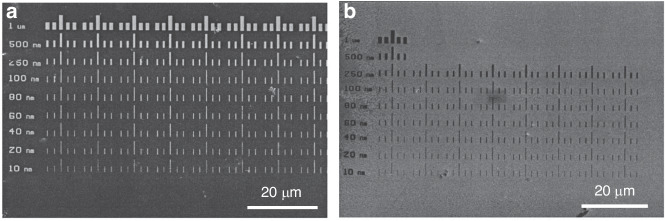
SEM images of the gold pattern generated by e-beam lithography and wet etching. **a** Biphenyl thiol (BPT) as a negative resist. **b** Hexadecane thiol (HDT) as a positive resist^27^. Reproduced with permission from AVS (2000)

Figure 32 shows gold line patterns varying from 10 nm to 1 µm, which were first obtained by e-beam lithography using biphenylthiol (BPT) (Fig. 32a) and hexadecanethiol (HDT) (Fig. 32b) and then fabricated by KCN/KOH wet etching for 45 min^27^. According to Fig. 32a, BPT was a kind of aromatic SAM, and the gold pattern underneath was protected due to crosslinking after e-beam exposure. In contrast, when HDT was used in Fig. 32b, it served as a positive resist, and the area exposed by the e-beam was etched away.

#### SAMs as e-beam resists on silicon

**Fig. 33:**
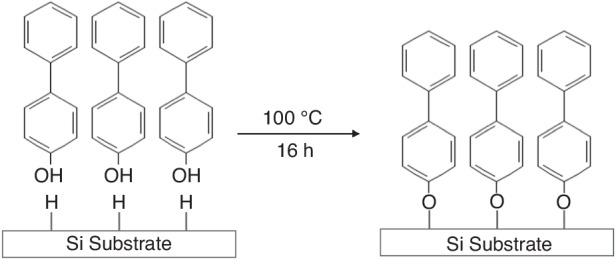
Schematic for the binding of hydroxybiphenyl (HBP) negative resist on silicon

Unlike gold, the formation of SAMs on silicon follows a different mechanism. The hydroxy group (-OH) usually connects SAMs and Si substrates. In other words, the same results as those in Section 4.1 can be obtained by changing the head group from -SH to -OH. E-beam lithography on Si using aromatic SAMs was achieved by Kuller et al.^81,82^. Figure 33 shows the formation of this kind of SAM. First, hydrogenated Si was prepared by soaking the wafer in H_2_SO_4_/H_2_O_2_ solution (3:1) and washing it in 48% HF. This wafer was immersed in 0.05 M 4-hydroxy-1,1′-biphenyl (HBP) in toluene and placed in a nitrogen atmosphere at 100 °C for 16 h. After the treatment, SAMs were formed on all the Si surfaces, including the nonplanar surfaces, because the SAM growth in the solution was isotropic. However, growing SAMs in solution has significant drawbacks, such as complicated process control, high demands for operator expertise and care, and high costs because the solution must be fresh and applied immediately before etching the coating^83,84^.

**Fig. 34:**
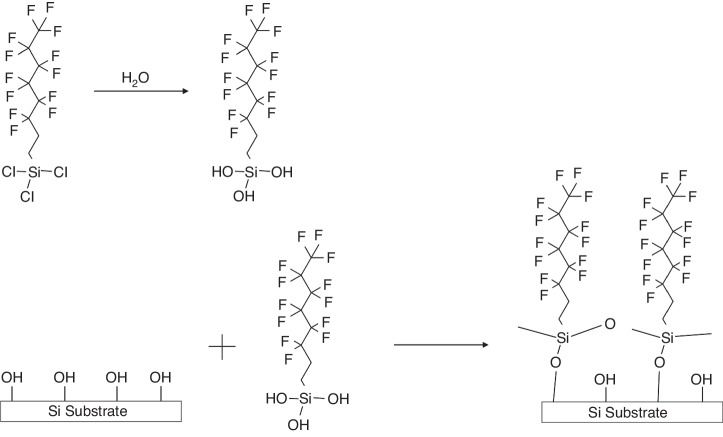
Mechanism of SAM formation on the hydroxylated silicon substrate

In addition to the liquid-phase growth of SAMs, vapor-phase growth has attracted an increasing number of researchers since it solves several problems associated with liquid-phase growth. This method eliminates the need for solutions and is thus easy to handle. In addition, the stoichiometry of the precursor can be easily controlled^83,84^. CF_3_(CF_2_)_5_(CH_2_)_2_SiCl_3_ (FOTS) and CF_3_(CF_2_)_7_(CH_2_)_2_SiCl_3_ (FDTS) are commonly used as precursors in the vapor-phase growth of SAMs^84^. The formation of FOTS or FDTS involves two steps, as demonstrated by Zhuang et al.^84^. In the first step, the head group reacts with water vapor to form the OH group, which bonds FOTS and FDTS to the silicon surface in the second step (Fig. 34). For small-scale coatings, such as those in which a single wafer is coated in the laboratory, the process can be simplified by placing a droplet of FOTS solution in a sealed beaker or a desiccator next to one Si wafer. After several hours, the droplet fully evaporates, and FOTS covers the entire wafer surface. This process is applicable to irregular surfaces since the evaporated FOTS vapor can occupy all the space and cover all the surfaces, whether flat or not.

To summarize, SAMs, as e-beam resists, are similar to grafted PMMA and PS because they form a chemically bonded monolayer. The unique growth mechanisms of SAMs make them well suited for surfaces with significant depth variations. Nonetheless, SAMs are too thin to resist dry etching, and wet etching is typically used for pattern transfer, resulting in subpar critical dimension control.

### E-beam lithography using thermally grown silicon dioxide resists

E-beam lithography using silicon dioxide as a resist, called e-beam stimulated oxide etching, was first recognized by O’Keeffe and Handy^85^. The scholars found that thermally grown silicon dioxide can be utilized as an e-beam resist since thermal SiO_2_ exposed to an e-beam (1–15 keV) has a relatively high etching rate in hydrofluoric acid (HF) solution. Compared to that of popular e-beam resists such as PMMA, the dose required to expose thermal SiO_2_ is much greater (approximately 5 orders of magnitude greater)^86^. Because the SiO_2_ resist is thermally grown and can fully cover all the sample surfaces, it can be used to pattern irregular and small surfaces. Si is the only substrate material applicable to thermal SiO_2_ growth, but it is very useful in some specific fields, such as the fabrication of silicon nanowires (SiNWs)^28,87^.

**Fig. 35:**
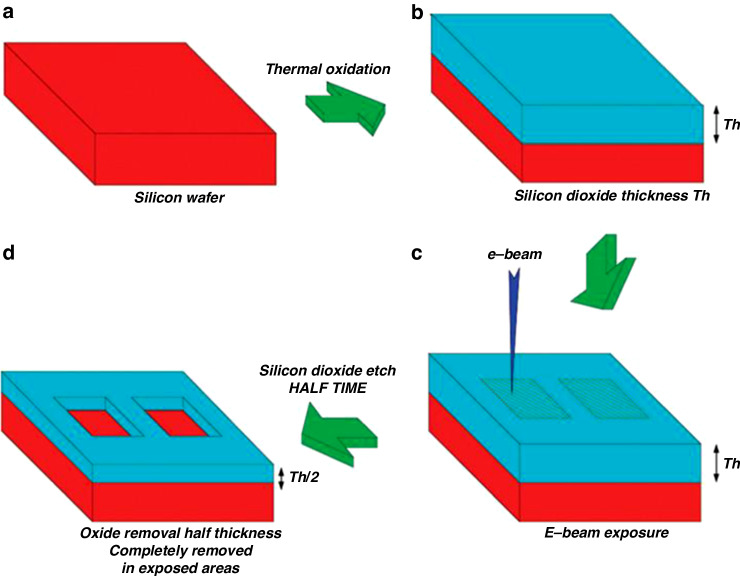
Schematic view of the e-beam stimulated oxide etching technique. **a** The starting substrate is a silicon wafer in the 〈100〉 orientation. **b** A silicon dioxide layer with a thickness of T_h_ is grown by thermal oxidation. **c** E-beam lithography is used for exposing delimited areas with a suitable dose. **d** Silicon dioxide wet etching (buffered HF) is performed for half the time required to remove the whole SiO_2_ layer^28^. Reproduced with permission from ACS Publications (2012)

**Fig. 36:**
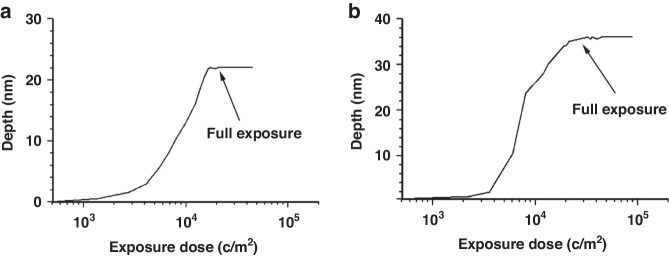
The depth of exposed areas is reported as a function of the exposure dose. **a** an initial layer 45 nm in thickness was used; after completing the half-time BHF etching process (in this case, 29 s), a 22-nm-thick layer of SiO_2_ remained in unexposed areas. **b** an initial layer with a thickness of 70 nm was used. The dose required for complete exposure was quite similar in the two cases^28^. Reproduced with permission from ACS Publications (2012)

The process flow of e-beam-stimulated oxide etching is shown in Fig. 35^28^. A SiO_2_ layer with a thickness of T_h_ was grown on a < 100> Si substrate, followed by exposure to the desired areas. Then, the sample was soaked in a buffered HF (BHF) solution^88^ (15 mL of H_2_O, 164 mL of 40% NH4F, and 22 mL of 48% HF). The etching rate of unexposed SiO_2_ was 50 nm/min. Optimally, e-beam-stimulated oxide etching should be performed at the half thickness (T_h_/2) of the unexposed area left while removing the exposed area. To achieve this goal, the exposed area depth as a function of dose was studied (Fig. 36)^28^. In case 1 (Fig. 36a), for T_h_ = 45 nm, the half etching time was 29 s. The exposed area was fully removed when the depth reached 22 nm after half etching. Figure 36b shows another case with a relatively thick oxide layer. Interestingly, both cases resulted in a similar full exposure dose (2 × 10^4 ^C/m^2^), suggesting that the initial oxide thickness did not significantly change the minimum dose of full exposure.

**Fig. 37:**
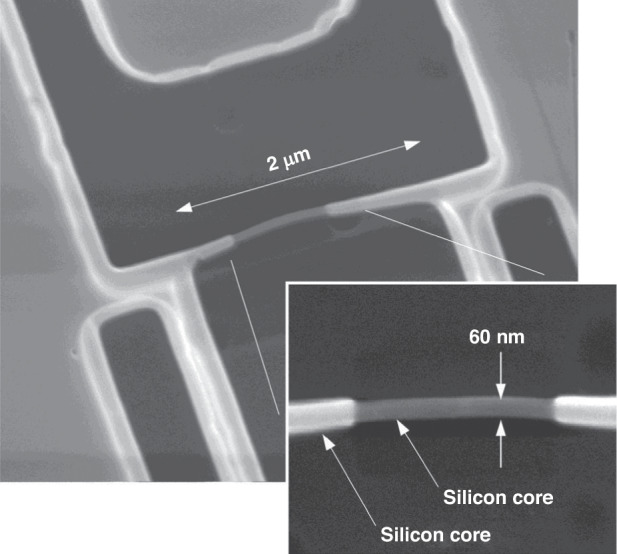
SEM images of a silicon nanowire. The silicon core was stripped from the oxide that still surrounds all the rest of nanowires^28^. Reproduced with permission from ACS Publications (2012)

Conducting e-beam stimulated oxide etching one time allows for the fabrication of silicon nanowires (SiNWs). SiNWs can be obtained by a top-down approach, in which multiple thermal oxidation and BHF etching steps are applied to reduce the width of the nanowires^89,90^. In the end, the silicon nanowire core is covered by SiO_2_ formed in the last oxidation step. Pennelli et al.^28^ followed a process that was previously reported^91,92^ to fabricate a SiNW device, selectively exposed the central area, and removed the oxide by HF etching. Figure 37 shows the SEM images of their results. The oxide in the central area (600-nm wide) was stripped by e-beam-stimulated oxide etching, while a T_h_/2-thick oxide layer remained in other areas. A 30% overexposure was applied to ensure that the SiO_2_ in the desired area was fully stripped.

## Conclusions and outlook

Electron beam lithography can increase the lithographic resolution to an unprecedented limit and allow for the patterning of nanostructures for a wide range of prototypical devices. The progress in coating resists on irregular or nonplanar samples has extended their ability to realize novel functionality. First, using thermal or electron-beam evaporation, sterol-based “dry” resists of QRS-5 and polystyrene were coated on arbitrary surfaces. After e-beam exposure and solvent-based development, lift-off and etching processes were performed to transfer patterns, such as focusing zone plates, on the tips of fibers and nanostructures on curved fiber surfaces. The cantilever features had resolutions on the order of tens of nanometers. Furthermore, the coevaporation of dry resists enabled the significant improvement in the etching selectivity from 1 to 33 by adding a small amount of Cr to polystyrene. In addition, 100-nm-wide and 3.5-µm-tall Si walls were achieved. The metal halide compounds NaCl, MgF_2_, LiF, and AlF_3_ could be coated by evaporation, and under high doses of e-beam exposure, the chains between metals and halogens were broken directly without development. Along with metal diffusion and halide dissociation, the e-beam exposure of metal halides resulted in extremely fine structures with sizes of 2 nm. Second, advances in ice e-beam lithography provided another possibility for patterning 3D structures on nonplanar substrates. Water and organic chemicals, as positive and negative resists, respectively, were pumped into cryostages and condensed to form uniform films on the samples. For the positive resist composed of water, the ice film was exposed, and the samples were transferred to another cryostage for material deposition. Pyramidal metal structures were formed on the AFM tips, and metal rings were wrapped around the nanotube. After 3 cycles of this process, even 3D metal structures could be achieved. Conversely, the negative ice resists composed of organic chemicals, such as C_5_H_12_O, C_7_H_8_O, C_8_H_18_, C_9_H_20_, C_11_H_24_, and C_14_H_30_, were crosslinked under electron beam exposure. The exposed samples were transferred to chambers at room temperature for etching and lift-off. Third, monolayers of grafted PMMA/polystyrene brushes were formed on irregular surfaces after resist coating, annealing, and solvent rinsing since a monolayer of PMMA or polystyrene was chemically bonded between the -COOH group in the PS layer and the -OH group in the Al layer. Grafted PMMA brushes retained the same characteristics as bulk PMMA samples and could be patterned with high resolution as both positive and negative resists. After e-beam exposure and development, Al wet etching and Si dry etching, a 30-nm-wide Si line array, and 14-nm-wide isolated lines were patterned on the edges and corners of the free-standing cantilevers of the AFM tips. PS could form a grafted brush on Al layers as a negative resist. This brush formation process was followed by etching. Fourth, similar to grafted brushes, SAMs were formed on Au surfaces through the -SH group of BPT and HDT molecules. However, on Si surfaces, SAMs of FOTS or FDTS were formed through the -OH group. The molecules of these SAMs could be broken or crosslinked by electron exposure. Since thin SAMs could significantly suppress electron scattering in the resist, fine nanostructures with sizes of 10 nm were patterned. In addition, thermal oxides acted to resist irregular surfaces, and after high-dose electron beam exposure, the wet etching rate doubled. The patterned oxide was stripped from Si nanowires to functionalize the nanostructures.

Notably, each of the aforementioned methods for irregular surfaces has its limitations. For example, evaporation of dry resist requires dedicated tools; water/organic ices must be handled in several cryostages for deposition and exposure. Both SAMs and grafted PMMA/PS brushes are very thin, with sizes of several nanometers, making pattern transfer difficult. Metal halide resists cause ion contamination in further processes and final devices. Moreover, the exposure dose for thermal oxides is 10^6^ times greater than that of commonly used PMMA or ZEP520 resists, resulting in small-area exposure. The results of these studies can be considered to surpass the limitations of the traditional e-beam lithography method on flat surfaces. These researchers offer new alternatives for creating patterns on uneven and nonplanar surfaces and allow for the fabrication of 3D nanostructures on rough surfaces, such as nanotubes, Si wires, cantilevers, and prepatterned 3D surfaces. E-beam lithography on nonplanar or irregular surfaces and subsequent pattern transfer technologies are widely used to fabricate prototype devices. These technologies have accelerated research in various applications, such as TERS, magnetic force microscopy, fiber optics, chemical and biological sensing, and quantum mechanical systems. The emerging requirements have triggered the development of new instrumentation, such as a built-in resist coating system and a dynamic electron beam focusing system.
